# Effectiveness of Aspirin on Double Lumen Permanent Catheter Efficacy in ESRD

**DOI:** 10.5812/numonthly.8733

**Published:** 2013-03-30

**Authors:** Mohammad Mozafar, Majid Samsami, Mohammad Reza Sobhiyeh, Sayena Jabbehdari, Mahtab Fallah Zavareh

**Affiliations:** 1 Shohada-e Tajrish Hospital, Shahid Beheshti University of Medical Sciences, Tehran, IR Iran; 2 Students’ Research Committee, Faculty of Medicine, Shahid Beheshti University of Medical Sciences, Tehran, IR Iran

**Keywords:** Aspirin, Catheters, Kidney Failure, Chronic Effects

## Abstract

**Background:**

The complications of vascular access are the most imperative etiology for hospitalization, morbidity and mortality in chronic hemodialysis. The most prevalent complication of central catheter is dysfunction due to thrombosis. Aspirin is an anti-aggregative platelet drug that may increase the patency of permanent catheters (perm-cath).

**Objectives:**

The aim of this study was to evaluate the role of Aspirin in perm-cath survival.

**Patients and Methods:**

This study included a total of 185 ESRD cases according to the inclusion criteria for perm-cath insertion in hemodialysis. One hundred and eighty patients following perm-cath insertion had proper blood flow through perm-cath during hemodialysis. Patients were randomly divided between intervention (80 mg/day Aspirin initiated a day following catheter insertion) and control (placebo) groups. The average time that the perm-cath was functional was noted. Demographic characteristics included comorbidities and past history were also used to address probable influence on perm-cath function and patency.

**Results:**

The mean survival time of the catheter in Aspirin group was significantly higher than the control group (5.3 ± 4.7 month versus 3.9 ± 2.7 month, P = 0.012). No significant difference in major complications of Aspirin use (such as GI bleeding) was noted between two groups (P = 0.52). In terms of the patient’s demographic characteristics, those of the female gender and a history of diabetes mellitus were found to have significant influence on median survival rate of the catheters (P = 0.021, 0.043 respectively).

**Conclusions:**

These results suggest that Aspirin use following perm-cath insertion might be beneficial for catheter survival. This increased survival time might enable patient’s use of AVF maturation for long term dialysis access.

## 1. Background

Hemodialysis catheters play an important role in the delivery of hemodialysis to patients for both temporary vascular and permanent accesses in patients with long term end-stage renal disease (ESRD). During the past decades, there has been an emergence of technological advancements in the design of dialysis catheters in an attempt to reduce catheter malfunction, decrease infection rates, and improvement of long-term efficiency. New improvements in catheter design have led to much needed improved catheter performance. Vascular access complications are the most important causes in hospitalization and morbidity in ESRD patients and the success of hemodialysis depends on permanent catheter (perm-cath) patency and function (1, 2). Furthermore, thrombosis is the most common complication of vascular access (2), and perm-cath use for hemodialysis. The annual cost of vascular access complications in US almost calculated one billion dollars (3). There is no cost analysis data available about vascular access complications in Iran. Heparinization can reduce thrombosis in catheters however this procedure is also subject to complications. Prevention of these kinds of complications is very important and should be noticed when the catheter is first inserted. Former studies have showed the effect of Warfarin in intravenous catheter perpetuity (4, 5). The other studies showed comparable effects from Aspirin in the dialysis catheter (6).

## 2. Objectives

The purpose of this study was to evaluate Aspirin’s effect on catheter survival in chronic renal failure patients needed regular dialysis. The use of oral anticoagulant and/or antiplatelet agents as primary or secondary prevention of tunneled catheter dysfunction must be weighed against their potential adverse effects, and should be individualized for each patient (7).

## 3. Patients and Methods

From June 2009 to June 2010, 185 dialysis patients arrived at Shohada-e-tajrish hospital’s surgical clinic, where this study was performed. This study included hemodialysis patients for whom arteriovenous access may be problematic, impossible, or delayed until arteriovenous access maturation for dialysis. Perm-cath insertion was performed by an experienced vascular surgeon. In five patients, there was poor blood flow following perm-cath insertion during hemodialysis, thus these patients were not included. Patients who had absolute contraindication to Aspirin were also excluded. The remaining patients were divided randomly into two groups of 90 patients. Underlying data such as age, sex, etiology of chronic renal failure, synchronic disease such as diabetes mellitus, hypertension, history of CVA (cerebrovascular disease) and IHD (ischemic heart disease), peripheral vascular disease, history of using Statin and anticoagulant medications and history of disadvantaged perm-cath insertion were asked for their probable effect assessment in two groups. Ninety ESRD patients that were in the case group were given Aspirin 80 mg/day on the first day following perm-cath insertion treatment. Indication or contraindication of Aspirin use was approved by a nephrologist in a double blind manner. The other 90 ESRD patients that were in the control group were administered a Placebo. An experienced vascular surgeon attended to a dysfunctional perm-cath that needed reinsertion since it had not received enough blood flow for acceptable hemodialysis and also evaluated a systemic infection due to a perm-cath’s bacteriemia. Quantitative data revealed by mean and qualitative data showed by percent and data was analyzed by SPSS version 17.0. For analyzing the quantitative variables t-test were operated and chi-square and Fisher’s exact tests were used for analyze qualitative variables. Statistical significant levels in this study were P < 0.05.

## 4. Results

From the 185 ESRD enrolled cases in this study, five patients were excluded due to perm-cath dysfunction. The other patients were surveyed, included 101 males (56%) and 79 females (44%). Fifty five males and 35 females took Aspirin and 53 males and 37 females were in the control group that had a placebo. All of patients were over 50 years old and the average age was 60 years old. In the group with the Aspirin prescription the average age was 60 ± 1 year however in the placebo group was 61 ± 1 year. Table 1 reveals the demographic data between the two study groups that there is no significant difference between these two groups in terms of preventable confounding variables. The primary cause of renal failure (ESRD) in the patients was diabetes mellitus (73.1%) however the second cause was HTN (69.7%). This study demonstrated perm-cath survival in prescribed Aspirin group which was significantly more than in the control group (5.3 ± 4.7 months compare with 3.9 ± 2.7 months P = 0.012); also in the male patients perm-cath survival rather than female cases (4.6 ± 0.4 months compared with 4.49 ± 0.2 months P = 0.021). In the diabetic patients, perm-cath survival was significantly less than patients without diabetes mellitus (4.7 ± 1.1 months compare with 4.3 ± 1.5 months P = 0.043). Other underlying variables such as peripheral vascular disease history had no effect on perm-cath survival. Complications of using Aspirin such as GI bleeding in melena form, hematemesis and any incidental findings during endoscopic that demonstrate GI bleeding was seen in the patients with Aspirin prescription (32%) and 27% in the control group however there was no significant difference between two groups (P = 0.52).

**Table 1.** Demographic Characteristics of Two Groups

Variable	With Aspirin (n = 90)	Without Aspirin (n = 90)	P value
Age, y (mean ± SD)	60 ± 1	61 ± 1.3	0.72
Male (%)	61.1	58.9	0.76
BMI, kg / m^2^(mean ± SD)	25.9 ± 2.2	30.1 ± 1.8	0.03
Perm-Cathdysfunction history (%)	3.3	1.1	0.6
CAD^a^ (%)	22.2	21.2	0.86
Heart failure (%)	41.1	36.7	0.54
Peripheral vascular disease (%)	12.2	11.1	0.82
HTN^a^more than 130/80, mmHg in two visits (%)	58.9	61.1	0.76
History of stroke (%)	0	0	1
DM^a^ (%)	77.8	75.6	0.72
History of cancer (%)	0	1.1	1
Historyof statin intake (mean ± SD)	3.9 ± 0.7	4.0 ± 0.1	0.81
Historyof using new anti-platelet drug (mean ± SD)	21.1 ± 0.7	27.8 ± 0.1	0.67
Statin using (mean ± SD)	88.9 ± 0.5	93.3 ± 0.2	0.58
Heart valve disease (%)	1.1	0	0.51

^a^Abbreviations: CAD, coronary artery diseases; DM, diabetes mellitus; HTN, hypertention

## 5. Discussion

A central catheter in hemodialysis patients is frequently disturbed by thrombosis with prevalence of 63% (8). Thrombosis may be noted based on its location and mechanism of clot formation. Catheter dysfunction means inadequate blood flow for hemodialysis however 300 cc per minute is defined as an adequate blood flow (9). Moreover, thrombosis is the most common complication. The average longevity of catheter reported 73-84 days and thrombosis is important cause of dysfunction in most of studies (10). The etiology of thrombosis in catheter has not defined yet, however the cause of thrombosis has been defined only in limited cases. The most likely cause of thrombosis was clot forming only related to activated coagulation cascade due to blood flow disorder (11). Furthermore, the main reason of this blood flow disorder is the existence of thrombosis within catheter lumen or a clot that has surrounded the tip of the catheter (12). When the inadequate heparin is injected within the catheter lumen following dialysis, the blood comes into the catheter and thrombosis forms in the catheter lumen and blocks the catheter. It has been demonstrated that neointima and thrombosis formed by oxidative stress and inflammation are most important in vascular access damage (13). The recent study demonstrated that Aspirin is an anti-platelet aggregation drug that reduces oxidative stress (14) as well as inflammation process (15). Furthermore, regarding platelet dysfunction in chronic renal failure patients, some studies revealed that the aggregation of platelets increases in dialysis (16, 17). This process may be lead to clot formation in dialysis catheter. Therefore using an anti-platelet drug may be beneficial and prevent a clot formation. Our study outcome suggests the beneficial effect of Aspirin as an anti-platelet medication in catheter survival. The same outcome was seen in Obialo’s study (6). The widespread use of Aspirin is its use as a prophylactic medication in cardiovascular disease and has been emphasized as such in different clinical trials and meta-analysis (18). The end stage renal disease (ESRD) has a significant correlation with cardiovascular disease (19). Thus, using Aspirin may have a twofold effect in ESRD patients. The findings of our study demonstrated that factors such as female gender and diabetes mellitus can decrease the odds for perm-cath survival despite Liu’s study in which the male gender and diabetes have had no relation with arteriovenous fistula (AVF) survival (20). These diverse results can be caused by the different kinds of AVFs and synthetic catheter structures, since the pathophysiology of AVF and catheter disorders are different.

In the literature review published in Medline database to 2011, there were no studies about the effects of underlying diseases and cardiovascular risk factors in perm-cath survival. Aspirin side effects such as GI bleeding is the most important factor for physicians in prescribing the medication but in this study there was no significant difference between complications of Aspirin between two groups. Regardless, there are some studies that indicate Aspirin usage increases mortality risk (21). Our study demonstrates Aspirin usage can increase perm-cath survival without incurring any important complications, but more widespread and multicenter studies are recommended to support this contention.
